# Sesamol Induces Apoptosis-Like Cell Death in *Leishmania donovani*

**DOI:** 10.3389/fcimb.2021.749420

**Published:** 2021-10-28

**Authors:** Rahat Ali, Shams Tabrez, Sajjadul Kadir Akand, Fazlur Rahman, Atahar Husein, Mohd Arish, Ali S. Alqahtani, Mohammad Z. Ahmed, Mohammad Husain, Abdur Rub

**Affiliations:** ^1^ Department of Biotechnology, Jamia Millia Islamia (A Central University), New Delhi, India; ^2^ Department of Pulmonary and Critical Care Medicine, Mayo Clinic, Rochester, MN, United States; ^3^ College of Pharmacy, Department of Pharmacognosy, King Saud University, Riyadh, Saudi Arabia

**Keywords:** apoptosis, ROS, cell cycle, oxidative stress, *Leishmania donovani*

## Abstract

**Background:**

Visceral leishmaniasis (VL), caused by the protozoan parasite *Leishmania donovani* (*L. donovani*), is the most severe form of leishmaniasis. It is largely responsible for significant morbidity and mortality in tropical and subtropical countries. Currently, available therapeutics have lots of limitations including high-cost, adverse side-effects, painful route of administration, less efficacy, and resistance. Therefore, it is time to search for cheap and effective antileishmanial agents. In the present work, we evaluated the antileishmanial potential of sesamol against promastigotes as well as intracellular amastigotes. Further, we tried to work out its mechanism of antileishmanial action on parasites through different assays.

**Methodology:**

*In vitro* and *ex vivo* antileishmanial assays were performed to evaluate the antileishmanial potential of sesamol on *L. donovani*. Cytotoxicity was determined by MTT assay on human THP-1-derived macrophages. Sesamol-induced morphological and ultrastructural changes were determined by electron microscopy. H_2_DCFDA staining, JC-1dye staining, and MitoSOX red staining were performed for reactive oxygen assay (ROS), mitochondrial membrane potential, and mitochondrial superoxide, respectively. Annexin V/PI staining for apoptosis, TUNEL assay, and DNA laddering for studying sesamol-induced DNA fragmentation were performed.

**Conclusions:**

Sesamol inhibited the growth and proliferation of *L. donovani* promastigotes in a dose-dependent manner. It also reduced the intracellular parasite load without causing significant toxicity on host-macrophages. Overall, it showed antileishmanial effects through induction of ROS, mitochondrial dysfunction, DNA fragmentation, cell cycle arrest, and apoptosis-like cell death to parasites. Our results suggested the possible use of sesamol for the treatment of leishmaniasis after further *in vivo* validations.

## Introduction

Leishmaniasis is caused by the protozoan parasite *L. donovani*. It has three main clinical forms: visceral leishmaniasis (VL), cutaneous leishmaniasis (CL), and mucocutaneous leishmaniasis (MCL). *L. donovani* is a digenetic parasite. It stays in sand flies as well as in mammals, including human. It exists in two major forms, extracellular promastigote and intramacrophagic amastigote. The promastigote stage resides in the sand fly vector, though amastigote stage resides in macrophages. Promastigotes are elongated, flagellated, and motile, whereas amastigotes are aflagellated and oval. The sand fly injects the promastigote into the skin of the mammalian host while sucking the blood (Burza et al., 2018). In the gut of the sand fly, it gets transformed into the infective promastigote form. An estimated 0.7–1 million new cases of leishmaniasis are reported every year from nearly 100 endemic countries (Burza et al., 2018). More than 90% of the worldwide cases of VL are reported from only seven countries including Brazil, Ethiopia, India, Kenya, Somalia, South Sudan, and Sudan (Ghatee et al., 2020). Chemotherapy is the only option for the treatment of the disease, though it is associated with various drawbacks. The drugs used for the treatment of VL include pentavalent antimonial, liposomal amphotericin B, paromomycin, and miltefosine. These treatments have several limitations due to their non-specificity, toxicity, painful route of administration, and high cost (Sundar et al., 2019). Hence, there is a need to search for novel antileishmanial agents with affordable price, high efficacy, and least toxicity. One possible source for such affordable treatment lies in the use of natural compounds purified from medicinal plants. Yet, many natural compounds, namely, quercetin, plumbagin, withanolide, cynaroside, and clerodane diterpene, have been evaluated for their antileishmanial potential (Fonseca-silva et al., 2011; Kathuria et al., 2014; Awasthi et al., 2016; Chandrasekaran et al., 2016; Tabrez et al., 2021). Many chemically synthesized compounds were also reported to have appreciable antileishmanial activity (Bekhit et al., 2018; Mukherjee et al., 2020; Thompson et al., 2021). Substantial amount of literature showed that plant-based products are relatively safer as compared to current synthetic antileishmanial drugs. A screening program the by Drug for Neglected Diseases initiative (DNDi), Geneva, Switzerland, is underway to search the cost-effective antileishmanial drug with high efficacy and better safety index ([[NoAuthor]]).

*Sesum indicum* is used in different medicinal preparations traditionally (Geetha et al., 2015). Sesame oil from the seed of this plant contains sesamol (3, 4-methylenedioxyphenol), which is extensively used in the Indian traditional system of medicine (Siriwarin and Weerapreeyakul, 2016). Its scientific evaluation is already validated and published by several research groups (Geetha et al., 2015; Siriwarin and Weerapreeyakul, 2016). Sesamol induced apoptosis-like features in HepG2, hepatocellular carcinoma cell line through reactive oxygen species (ROS) generation, cell cycle arrest, DNA fragmentation, and mitochondrial dysfunction (Liu et al., 2017). Antibacterial, antifungal, anti-inflammatory, and immunomodulatory activities of sesamol have already been reported (Ansari et al., 2017; Hans et al., 2017). However, no study has been done so far regarding the antileishmanial activity of sesamol. In the light of previous findings of it, we planned to evaluate its antileishmanial potential. We found that sesamol exhibited dose-dependent abrogation in the growth and proliferation of promastigotes as well as intracellular amastigotes. Electron microscopy (SEM and TEM) further corroborated *in vitro* antileishmanial potential of this compound by inducing morphological and ultrastructural changes. To further elucidate the possible mechanism of action, we performed several assays including ROS generation, mitochondrial membrane potential, mitochondrial superoxide generation, DNA fragmentation, cell cycle arrest, and annexin V/PI assay. We observed that sesamol induced apoptosis-like cell death in *L. donovani* promastigote parasite through the generation of ROS and depolarization of mitochondrial membrane potential. Our results may be helpful to develop new natural compound-based antileishmanial drug having low cost, higher efficacy, and better safety index.

## Material and Methods

### Chemicals

M199 media, Roswell park memorial institute (RPMI) 1640 media, penicillin-streptomycin antibiotic cocktail, and Fetal bovine serum (FBS) were purchased from GIBCO (Life Technologies). Miltefosine and MTT reagents were purchased from Cayman Chemical and Merck, respectively. Propidium iodide (PI), Annexin-V/FITC (Fluorescein isothiocyanate) apoptosis kit, H_2_DCFDA dye, JC-1dye, MitoSOX red, and TUNEL assay kit were procured from Invitrogen (Thermo Fisher Scientific). Sesamol was purchased from Sigma Aldrich and dissolved in DMSO to yield a stock solution (10 mM).

### Parasite and Cell Culture

*L. donovani* AG83 strain was maintained freshly in M199 medium, supplemented with 10% heat-inactivated FBS and 1% streptomycin-penicillin antibiotic at 22°C. The culture was passaged periodically for 72 to 96 h until it reached its exponential phase. THP-1 monocyte cell lines were grown in RPMI 1640 medium having 10% heat-inactivated FBS and 1% streptomycin-penicillin at 37°C in the incubator with 5% CO_2_.

### Anti-Promastigote Activity

*L. donovani* logarithmic-phase-promastigotes (2×10^6^ parasites/ml) were incubated in 12-well plate in M199 media with different concentrations (6.2, 12.5, 25, 50, 100, and 200 µM) of sesamol for 48 h. Parasites were fixed in 1% paraformaldehyde, and viable parasites were counted through the Neubauer chamber with a coverslip. Untreated parasites served as the negative control. Half maximal 50% inhibitory concentration (IC_50_) was determined by plotting the graph between doses *versus* viability (Ali et al., 2021). IC_50_ value was determined by a dose-response curve using GraphPad Prism 6, and the graph was plotted between viability *versus* dose.

### Scanning Electron Microscopy

*L. donovani* promastigotes (2×10^6^ cells/ml) were incubated with a 2×IC_50_ dose of sesamol for 48 h. Post-treatment, the cells were further harvested, washed in 1×PBS (pH 7.4), and fixed in Karnovasky fixative (4% paraformaldehyde and 2% glutaraldehyde) overnight at 4°C. Afterward, cells were washed with 1×PBS, three times and dried in the air. The samples were further coated with a gold-palladium sputter coater and finally visualized in an LEO 435 electron microscope using an accelerating voltage of 20 kV.

### Transmission Electron Microscopy

*L. donovani* promastigotes (2×10^6^ cells/ml) were seeded in a six-well plate and incubated with a 2×IC_50_ dose of sesamol for 48 h. Post-treatment, parasites were further harvested and washed in 1×PBS (pH 7.4). Subsequently, cells were fixed in Karnovasky fixative (4% paraformaldehyde and 2% glutaraldehyde) overnight at 4°C. Thin sections were stained with uranyl acetate and lead citrate for visualization by LEO 435 Transmission electron microscope.

### Cytotoxicity Assay

Cytotoxic potential of sesamol was evaluated by MTT assay. THP-1 cells were seeded into 96-well plates at the density of 2×10^5^ cells/ml with supplementation of 5 ng/ml PMA. After differentiation into macrophages, cells were further treated with different doses of compounds. MTT reagent (3-(4, 5-dimethylthiazol-2-yl)-2, 5-diphenyl tetrazolium bromide) 20 µl/well (5 mg/ml in 1×PBS) was added in each well followed by incubation for 4 h at 37°C in CO_2_ incubator. In the post-incubation period, the media were discarded, and the yellow precipitate was further dissolved in 200 µl acidified isopropanol for 30 min to solubilize the formazan crystals. Spectrometric absorbance was read at 570 nm on ELISA reader (Thermo Scientific Varioskan). The 50% cytotoxic concentration (CC_50_) was calculated by a dose-response curve using GraphPad Prism 6. Cell viability graph was plotted between viability *versus* concentration. Selectivity index (SI) was determined by the ratio of the CC_50_/IC_50_ values.

### Anti-Amastigote Assay

To evaluate the anti-amastigote activity, 0.5 × 10^6^ THP-1 cells were plated on coverslips in a six-well plate. Cells were further allowed to differentiate into macrophages for 12 h. Afterward, differentiated macrophages were further infected with extracellular promastigote parasites in ratio 1:10 (macrophage:parasite) and incubated for 12 h at 37°C in 5% CO_2_. After 12 h, unbound parasites were removed, and infected cells were further incubated with varying concentration (200, 100, 50, 25, and 12.6 µM) of sesamol for 48 h. Post-treatment, cells were washed with 1×PBS, fixed in chilled methanol for 20 min, and further slides were stained with Giemsa for 30 min. Furthermore, slides were examined on a light microscope at 40× magnification. At least 100 macrophages were counted from the randomly selected region from each slide, and the infectivity index was calculated (Husein et al., 2018).

### ROS Production Assay

We used the fluorescent dye H2DCFDA to assess the ROS production. Then 2 × 10^6^ parasites/ml were incubated with different concentrations of sesamol as mentioned for 48 h. Post-incubation, treated parasites were harvested and washed three times in 1×PBS. Furthermore, 10 µM H2DCFDA dye in 500 µl 1×PBS for 30 min in dark at room temperature. Fluorescence intensity was measured with Attune NxT flow cytometer (Thermo Fisher Scientific) using excitation wavelength 488 nm (Islamuddin et al., 2014). The histograms were representative of three independent experiments, and a total of 10,000 events for all samples were taken to ensure adequate data.

### Mitochondrial Superoxide Production Assay

Mitochondrial superoxide production was estimated by fluorescent dye MitoSOX red. Then 2 × 10^6^ parasites/ml were treated with IC_50_ and 2×IC_50_ doses of sesamol for 48 h. Subsequently, parasites were harvested, washed with 1×PBS, and incubated with 5 µM MitoSOX red solution for 20 min at room temperature. The fluorescence intensity was measured by BD FACS Aria flow cytometer at the excitation wavelength of 514 nm and with a bandpass filter 575–615 nm for emission wavelength. A total of 10,000 events for all samples were taken to ensure adequate data. The histograms are representative of three independent experiments.

### Measurement of Mitochondrial Membrane Potential

Loss of electrochemical gradient of mitochondria is the key feature of apoptosis. Loss of mitochondrial membrane potential was measured by JC-1 potentiometric dye. Parasites (2 × 10^6^ parasites/ml) were incubated with IC_50_ and 2×IC_50_ doses of sesamol for 48 h. Post-incubation, cells were harvested and washed three times with 1×PBS. Subsequently, 10 µM JC-1dye was added for 20 min in the dark at room temperature. The ratio of 590/530 (red/green) fluorescence demonstrated the relative change in mitochondrial membrane potential (ΔΨ_m_). The samples were analyzed by Attune NxT flow cytometer (Thermo Scientific) using excitation wavelength 488 nm and emission filter with a bandpass of 505 to 550 nm (green) and long pass of 575 nm (red). Ten thousand events for each sample were taken to ensure adequate data. The dot plots are representative of three independent experiments.

### *In Situ* DNA Fragmentation by TUNEL Assay

DNA fragmentation assay was performed by using TUNEL assay kit (Invitrogen) as per the manufacturer’s protocol. Parasites (2×10^6^ parasites/ml) were incubated with sesamol for 48 h. Post-treatment, cells were washed, fixed with 4% paraformaldehyde for 1 h, and further washed with 1×PBS. Pellet was further permeabilized with chilled 0.1% Triton X-100 detergent for 5 min. Further, cells were washed twice with 1×PBS. After washing, cells were further incubated with DNA−labeling solution (TdT+ Brd UTP) for 60 min at 37°C in water bath. Subsequently, 5.0 μl antibody staining solution was added in each tube (Alexa Fluor™ 488 dye tagged with anti-BrdU antibody) and incubated for the next 30 min at room temperature. Cells were further washed and finally stained with PI/RNase A staining buffer in the dark for the next 130 min. Samples were analyzed in Attune NxT flow cytometer (Thermo Fisher Scientific) at excitation wavelength 488 nm with FITC/PI channel (Islamuddin et al., 2014). Ten thousand events for each sample were taken to ensure adequate data. The histograms are representative of three independent experiments.

### DNA Laddering Assay

Parasites (2 × 10^6^ parasites/ml) were lysed in 500 µl of lysis buffer (10 mM EDTA 50 mM, 0.5% SDS Tris-HCl, pH 7.5) supplemented with proteinase K (100 µg/ml) and allowed to digest overnight at 50°C. Further, RNase A (0.3 mg/ml) was added and incubated at 37°C for 1 h. The parasite lysates were then extracted by phenol-chloroform-isoamyl alcohol (25:24:1) and centrifuged at 15,000 × g for 10 min. The upper aqueous phase was treated with 3 M sodium acetate and 100% ethanol overnight at −20°C. Furthermore, the sample was centrifuged at 16,000 × g for 10 min, and the supernatant was removed and 500 µl of 70% ethanol was added. The DNA pellet was dissolved in TE buffer (10 mM Tris-HCl, 1 mM EDTA; pH 8.0). Spectrometric absorption was read at 260/280 nm at Spectrometer (Eppendorf). Total genomic DNA was separated on 1.5% agarose gel containing ethidium bromide. The gel was run for 1 h at 90 V and visualized by a UV illuminator (Genei).

### Flow Cytometric Analysis of Sub G_0/_G_1_ of the Cell Cycle

Parasites (2×10^6^ parasites/ml) were incubated with IC_50_ and 2×IC_50_ doses of sesamol and parasites without treatment taken as the negative control. Post-treatment, cells were harvested, washed twice with 1×PBS, and fixed with 70% ethanol at 4°C for 24 h. Now fixed parasites were further washed with 1×PBS and pellet supplemented with 500 µl RNase A (20 mg/ml) incubated at 37°C for 1 h. The parasite was further incubated with 50 µg/ml PI in the dark for 20 min. The percentage of cell count in G_0_, G1, S, and G_2_/M phases of the cell cycle was determined by BD FACS Aria flow cytometer Aria (Sultana et al., 2018). A total of 10,000 events for all samples were taken to ensure adequate data. The histograms are representative of three independent experiments.

### Apoptotic Assay

*L. donovani* promastigotes (2 × 10^6^ parasites/ml) were incubated with IC_50_ and 2×IC_50_ doses of sesamol. Post-incubation, treated and untreated promastigotes were harvested and centrifuged at 3,000 g for 10 min and washed twice in 1×PBS. Further, the pellet was resuspended in 195 µl of 1× binding buffer along with the addition of 5 µl Annexin V/FITC and 5 µl PI-containing solution. After 20 min of incubation in the dark at room temperature, the sample was analyzed by BD FACS Aria flow cytometry (Yousuf et al., 2018). The percentage of live and dead cells was determined using FITC/PI channel, and 10,000 events for each sample were acquired. The dot plots were representative of three independent experiments.

### Statistical Analysis

All values are expressed as Mean± SD. All experiments were performed in triplicate independently. Unpaired student’s t-test was applied to calculate significant differences of two groups, and P values <0.05 were considered as significant.

## Results

### Anti-Promastigotes Activity Evaluation of Sesamol (IC_50_)

**Figure 1:**
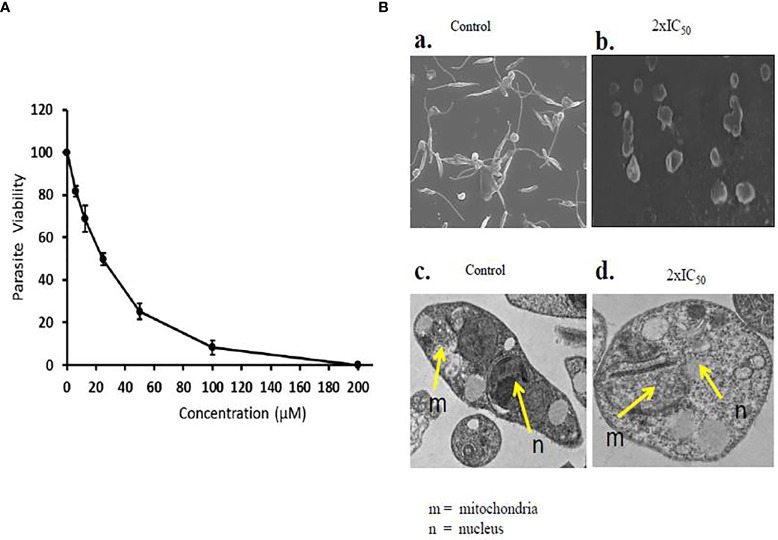
Antileishmanial effect of sesamol on *L. donovani* promastigotes. **(A)** *L. donovani* promastigotes were treated with a dose range of 0 to 200 µM for 48 h, and IC_50_ was calculated by graphical exploitation from GraphPad Prism 6. **(B)** SEM and TEM micrographs of morphological changes induced by sesamol in *L. donovani*; **(a)** SEM micrographs of untreated samples showing long flagella and elongated body, **(b)** SEM micrographs of sesamol with 2×IC_50_-treated promastigotes showed short flagella and round shape. Bars 5 µm. **(c)** TEM micrographs of untreated control having the normal structure of nucleus (n) in parasites and mitochondria (m)**. (d)** TEM micrographs of sesamol treated with 2×IC_50_ dose showing distortions in nuclear shape (n) and disorganization of mitochondria (m). Bars 1 µm. The graph represents the mean ± SEM of three independent experiments in triplicate.

Sesamol effectively inhibited the viability of promastigote parasites in a dose-dependent manner in comparison to untreated parasite control. A substantial amount of inhibition was observed at higher concentrations, and the parasite did not revert to normal shape post-incubation. At lower doses, sesamol showed moderate effect; meanwhile, nearly 90% growth inhibition was observed at 200 µM. IC_50_ value of the compound on parasite promastigote after 48 h was determined as 25.19 ± 1.44 µM **(** **Figure 1A**). The graph showed the value of three independent experiments along with standard deviation.

### Scanning Electron Microscopy and Transmission Electron Microscopy of Sesamol-Treated Promastigotes

Morphological changes and subcellular alterations are some of the key hallmarks of an early stage of apoptosis. Here, we found that sesamol altered the size, shape, and length of flagella at 2×IC_50_ concentration. Some of the parasites were observed as lysed under SEM. Though, untreated control parasites were observed with long flagella at the anterior end, having elongated bodies (**Figures 1** **Ba, Bb**). TEM images showed abnormal chromatin condensation with distorted shapes of the nucleus and mitochondria. The observed subcellular alterations indicated that mitochondria were adversely affected by sesamol treatment (**Figures 1** **Bc, Bd** **).** Similar results were observed with the essential oils of *Artemisia annua* on *L*. *donovani* promastigotes (Islamuddin et al., 2014).

### Anti-Amastigote Activity, Cytotoxicity (CC_50_), and Selectivity Index Calculation

**Figure 2:**
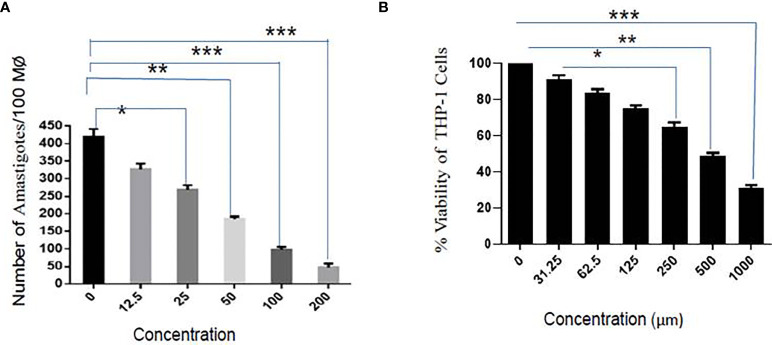
Effect of sesamol on *L. donovani* amastigotes. **(A)** Dose-dependent effect of sesamol on intramacrophagic amastigotes after 48 h of treatment. **(B)** THP-1 derived macrophages were treated with sesamol for 48 h, and further MTT assay was performed to assess the cytotoxicity. The graph represents the mean ± SEM of three independent experiments in triplicate. Significance level is *P < 0.05, **P < 0.01, and ***P < 0.001.

Parasite entry inside the macrophages involves the formation of membrane-bound parasitophorous vacuoles, where they differentiate into non-motile amastigote form. The parasite’s survival within the parasitophorous vacuole defines the pathogenesis of parasites. It has clinical relevance, and therefore, it is of paramount importance to test the activity of sesamol on intracellular amastigotes. The infectivity index exhibited a dose-dependent reduction in the number of amastigotes in treated infected macrophages with EC_50_ value of 35.56 ± 2.94 μM (**Figure 2A**). Sesamol treatment significantly reduced nearly 90% intracellular amastigotes at 200 μM concentration, and progressive reduction in the number of amastigotes represented as pink dots by Giemsa-stained micrograph. Though, we observed through the MTT assay that sesamol showed very low cytotoxicity on THP-1-derived human macrophages. The cytotoxic effect of the compound was evaluated by MTT assay after 48 h of treatment. It was observed that sesamol displayed a less cytotoxic effect on the morphology and viability of the macrophages with CC_50_ of 595.52 ± 1.64 µM (**Figure 2B**). Cytotoxic studies on human macrophage cell line (THP-1) showed that up to 400 μM, sesamol has no toxic effect on the human macrophages cell line. The toxicity for THP-1-differentiated macrophage was compared with activity against *L. donovani* parasites by calculating selectivity index (CC_50_ for THP-1 cell/IC_50_ for the parasite), about 23.6 and 16.7 **(** **Table 1** **)** on extracellular promastigotes and intracellular amastigotes, respectively. Our results demonstrate that sesamol is selective against parasites without showing undesirable effects on human macrophages. Natural compounds having selectivity index values greater than 10 were generally considered as promising lead molecules.

**Table 1:** Selectivity index calculation.

Parasite	Drug	CC_50_	IC_50_	SI Index
Promastigote	Sesamol	595.52 µM	25.19 µM	23.6
Amastigote	Sesamol	595.52 µM	35.56 µM	16.7

### Sesamol Induced Oxidative Stress in *L. donovani* Promastigotes Through ROS

**Figure 3:**
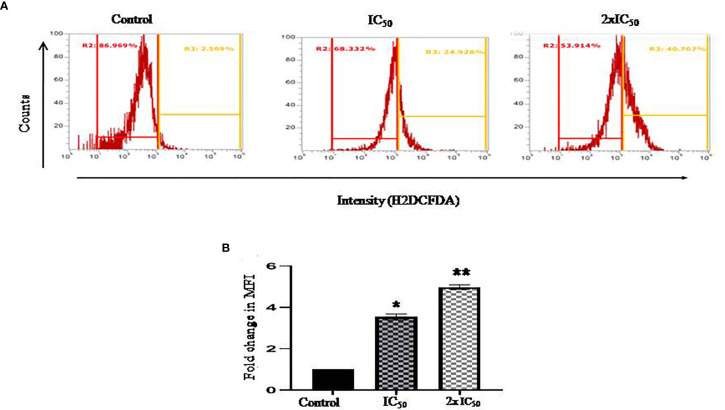
Sesamol-induced ROS generation in parasites. Sesamol induced the production of ROS in a dose-dependent manner in *L. donovani* promastigotes after 48 h of sesamol treatment through H2DCFDA staining as shown by **(A)** flow cytometric analysis, **(B)** Mean fluorescence intensity plotted *vs* concentration of sesamol. Flow cytometric histogram represented three independent experiments. The graph represents the mean ± SEM of three independent experiments in triplicate. Significance level is *P < 0.05, **P < 0.01.

ROS induction plays an instrumental role in various oxidative stress-related cellular processes, which regulate the fate of the cell. H_2_DCFDA staining was performed to evaluate sesamol-induced ROS generation in parasites. In our work, we found a significant shift of fluorescence in the right direction at IC_50_ and 2×IC_50_ concentrations. It was found that the treatment of sesamol induced ROS production in a dose-dependent manner in parasites. We observed 24% ROS-positive and 40% ROS-positive parasites upon sesamol treatment at IC_50_ and 2×IC_50_ doses, respectively (**Figures 3A, B** **)**. From these findings, it could be inferred that sesamol possibly induced oxidative stress through ROS generation. Similar findings were reported by the previous investigator (Afrin et al., 2019).

### Sesamol Induced Depolarization of Mitochondrial Membrane of Parasite

**Figure 4:**
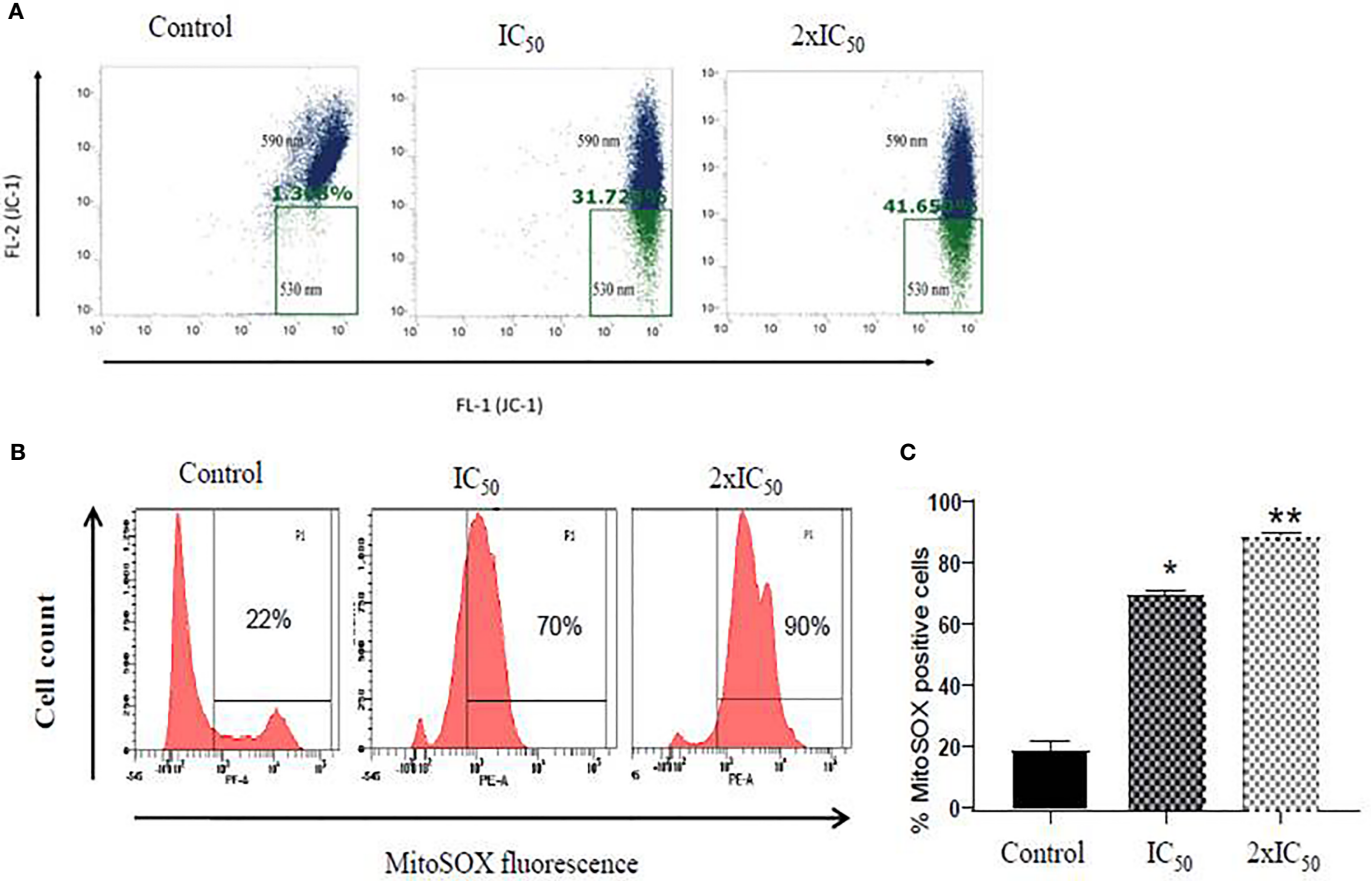
Sesamol induced depolarization of mitochondrial membrane and superoxide generation. **(A)** Dot plot showed shifting of fluorescence in the green channel (530 nm, FL-1). A significant decrease in red/green (590/530 nm) intensity further indicated depolarization of mitochondrial membrane potential treated with IC_50_ and 2×IC_50_ doses of sesamol using JC-1 dye. **(B)** Mitochondrial superoxide generation in *L. donovani* promastigotes using fluorescent dye MitoSOX red at IC_50_ and 2×IC_50_ doses of sesamol. **(C)** Bar graph representing mean % of superoxide positive cells. Flow cytometric histogram represented three independent experiments. The graph represents the mean ± SEM of three independent experiments in triplicate. Significance level is *P < 0.05, **P < 0.01.

Maintenance of electrochemical gradient across the mitochondrial membrane is important for cell death and survival of the cell (Kathuria et al., 2014; Mukherjee et al., 2020). Flow cytometric analysis of sesamol-treated parasites with JC-1 potentiometric dye demonstrated a steady and dose-dependent increase in the number of depolarized mitochondria of parasites. We observed 31 and 47% of mitochondria are depolarized at IC_50_ and 2×IC_50_ doses of sesamol after 48 h with respect to untreated control **(** **Figure 4A** **)**. Our data demonstrated that sesamol induced a remarkable decrease in red/green (590/530) fluorescence intensity ratio, which indicated depolarization of the mitochondrial membrane (Chouhan et al., 2015). Mitochondrial dysfunction is an established feature of apoptosis-like cell death and which possibly led to DNA fragmentation.

### Sesamol Induced Mitochondrial Superoxide Anion Generation in *L. donovani* Parasite

As sesamol induced the membrane depolarization of mitochondria of parasites, therefore, we further planned to study the generation of superoxide radicals formation in it. It was measured by MitoSOX red staining through flow cytometric analysis. Sesamol increased the superoxide anions in a dose-dependent manner in parasites **(** **Figure 4B** **)**. It was found that 70 and 90% of parasites having positive for superoxide radicals at IC_50_ and 2×IC_50_ doses, respectively **(** **Figures 4B, C** **)**.

### Sesamol Induced DNA Fragmentation

**Figure 5:**
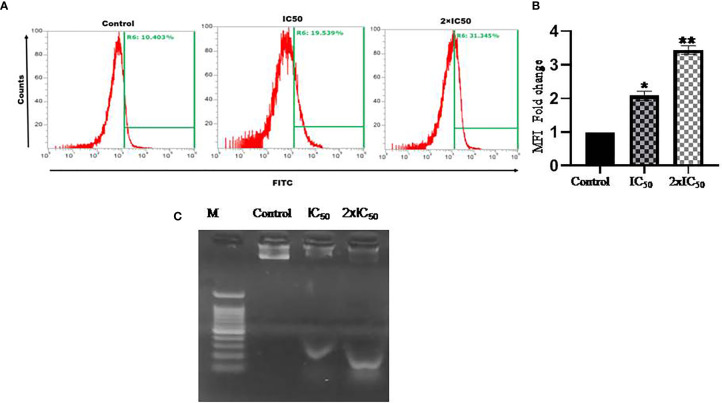
Sesamol-induced DNA fragmentation in the parasite. **(A)** Flow cytometric analysis of sesamol-induced DNA fragmentation was observed through TUNEL assay for 48 h of treatment at IC_50_ and 2×IC_50_ concentrations. **(B)** Mean of TUNEL +ve cells represented as a bar graph. **(C)** *L. donovani* genomic DNA fragmentation assay. Lane M. 1 kb ladder, treated with IC_50_ in Lane 2 and 2×IC_50_ in Lane 3 showing sesamol-induced DNA fragmentation. The histograms are the representative images of three independent experiments. The graph represents the mean ± SEM of three independent experiments in triplicate. Significance level is *P < 0.05, **P < 0.01.

Cleavage of genomic DNA into its small fragments is a characteristic feature of a cell dying through apoptosis mode of cell death. Sesamol-treated and untreated parasites were analyzed by flow cytometry and revealed that TUNEL^+^ parasites were increased with increasing doses of treatment. Around 24.9 and 40% of parasites were TUNEL^+^ at IC_50_ and 2×IC_50_ doses of sesamol respectively **(** **Figures 5A, B** **).** These findings indicated that sesamol treatment induced DNA fragmentation of *Leishmania* promastigotes, which further mediated the apoptotic mode of cell death.

### DNA Laddering Assay

DNA laddering assay was performed to further validate the findings of TUNEL assay. Sesamol induced the significant fragmentation in genomic DNA of the parasites as observed on agarose gel **(** **Figure 5C** **)**. There was a marked genomic DNA fragmentation at IC_50_ dose (lane 2) and 2×IC_50_ dose (lane 3) of sesamol. The results clearly showed that cell death induced by sesamol was most likely due to the activation of endonucleases upon sesamol treatment.

### Sesamol Triggered Cell Cycle Arrest of Parasites in G_0_/G_1_ Phase

**Figure 6:**
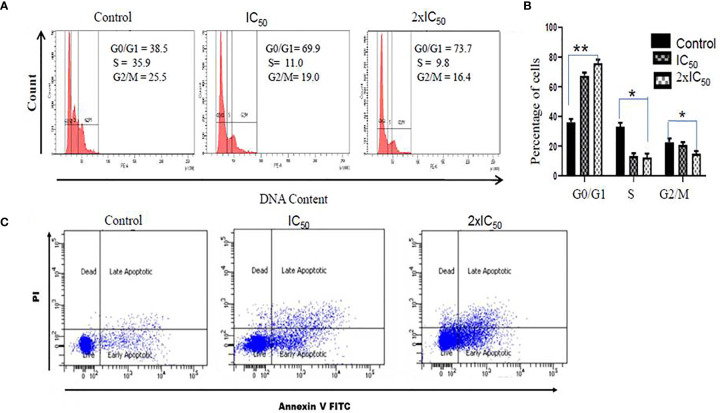
Sesamol induced cell cycle arrest and induction of apoptosis-like cell death in *L. donovani* promastigotes. **(A)** Sesamol treatment arrested the cell cycle in the G_0_-G_1_ phase of the cell cycle as analyzed by flow cytometry. Flow cytometric histogram represented three independent experiments. **(B)** Mean % of different phases of the cell cycle as represented by bar graph. **(C)** Dot plot showed that at IC_50_ and 2×IC_50_ doses, sesamol induced phosphatidylserine externalization, studies by annexin V/PI staining. The graph represents the mean ± SEM of three independent experiments in triplicate. Significance level is *P < 0.05, **P < 0.01.

To evaluate the effect of sesamol on the cell cycle regulation of parasites, we performed PI staining. Interestingly, it was found that the sesamol treatment modulated the progression of the cell cycle in parasites **(** **Figures 6A, B** **)**. PI staining of untreated parasites showed 38.5% of parasites in the G_0_/G_1_ phase of the cycle. It was observed 69.9 and 73.7% parasites in the G_0_/G_1_ phase of the cell cycle at IC_50_ dose and 2×IC_50_ dose, respectively **(** **Figure 6B** **)**. There was no significant change observed in the G2/M phase of the cell cycle upon sesamol treatment. Our data suggested that sesamol induced the arrest of parasites in the G_0_/G_1_ phase of the cell cycle in a dose-dependent manner. This arrest of parasites in G_0_/G_1_ is also an event of apoptosis-like cell death (Sultana et al., 2018).

### Sesamol-Induced Phosphatidylserine Externalization in Parasite Membrane

Translocation of phosphatidylserine (PS**)** from the inner leaflet to the outer leaflet is the established feature of apoptosis. Annexin V, a Ca^2+^-dependent protein, has a high affinity to phosphatidylserine (PS**)** and is used for qualitative analysis of apoptosis. Here, we observed 18.4% parasites in early apoptosis and 8.9% parasites in late apoptosis at IC_50_ dose. But at the 2×IC_50_ concentration, significant shifts of cells were observed in early apoptotic cells (33.6%) and late apoptotic cells (25.5%) **(** **Figure 6C** **)**. These results strongly supported that sesamol-induced apoptosis-like cell death was mediated through externalization of PS in *L. donovani* promastigote cell membrane.

## Discussion

The current treatment that is available for VL has many limitations due to their side effects, low efficacy, high cost, and high toxicity. Previously, various pathways had been targeted for the identification of antileishmanial lead molecules by us and others to combat this menace (Rub et al., 2009; Kathuria et al., 2014). For the last few years, there has been a trend of revival of the usage of plant and plant-derived natural products for the treatments of various infectious diseases globally. One of them is polyphenolic sesamol, the major constituent of the sesame seed of plant *Sesum indicum.* It has been used in alternative systems of medicine for the last several years (Ansari et al., 2017; Hans et al., 2017; Liu et al., 2017). We assessed the half-maximal 50% inhibitory concentration (IC_50_) and half-maximal 50% cytotoxic concentration (CC_50_) of sesamol to evaluate its antileishmanial potential. We observed that it inhibited the growth of promastigotes in culture in a dose-dependent manner. Actually, ultimate targets of antileishmanial drugs are to kill the intracellular amastigotes which are associated with disease progression in the human host. Therefore, we tested it on infected macrophages. We observed dose-dependent anti-amastigotes activity with IC_50_ value of 35.56 ± 2.94 μM. At 200 μM concentration, it reduced the intracellular parasite burden up to more than 80%. Cytotoxic studies on THP-1-derived human macrophages showed that sesamol had least cytotoxic effect up to 400 μM concentration. Selectivity index (SI) was observed in a good and under permissible limits for a lead molecule. Scanning Electron Microscopy images showed that sesamol has a profound effect on parasites morphology. Sesamol induced many types of morphological alterations in parasite shape and size such *viz.* rounding of cells, cell shrinkage, loss of flagella, and reduction in length. TEM results indicated ultrastructural alterations like nuclear condensation, disorganized mitochondrial structure, and intense cytoplasmic vacuolation. Similar effects in morphological and ultrastructural alterations were observed with earlier studied natural lead molecules like plumbagin and yangambin (lignin) on promastigotes (Neto et al., 2011; Awasthi et al., 2016). Quercetin and withaferin induced apoptosis-like cell death by generating ROS promastigotes form (Kathuria et al., 2014; Afrin et al., 2019). We also observed ROS production in a dose-dependent manner in parasites. Mitochondria, a vital organelle, is involved in many signaling pathways and fundamental cellular processes. Earlier studies showed the disturbance in the mitochondrial structure and function would lead to parasite cell death (Sen et al., 2004). Sesamol eventually caused mitochondrial dysfunction through excessive ROS generation. A higher ROS generation inside parasites has detrimental effects on them. We observed remarkable decrease in red/green (590/530 nm) fluorescence intensity of JC-1 potentiometric dye, which suggested the collapse of mitochondrial membrane polarization, which would lead to the stress in parasites causing apoptosis-like cell death. Previously, many investigators had also shown a similar mechanism of action of several natural compounds showing antileishmanial activity (Mukherjee et al., 2020; Mustafa et al., 2021). In multicellular organisms, mitochondria produced superoxide anions and free radicals upon exposure to toxicants, which subsequently oxidized and damaged lipids, proteins, and DNA of the cells (Shadab et al., 2017). Here, we found that sesamol significantly generated free radicals and O_2_^-^ in *L. donovani* promastigotes.

To further dissect the effect of ROS and superoxide generation in parasites, we decided to perform DNA fragmentation assay. Previous work had reported that several natural products induced DNA nicks in the parasite’s genomic DNA (Paris et al., 2004; Shadab et al., 2017). Cleavage of genomic DNA into its nucleosomal components is a characteristics feature of cell dying through apoptosis (Paris et al., 2004; Afrin et al., 2019). We found a progressive increase of TUNEL^+^ cells in a dose-dependent manner in comparison to untreated control. Qualitative analysis of DNA fragmentation by DNA laddering assay unequivocally supported the findings of TUNEL assay. This result is consistent with previous studies by other groups on different natural lead molecules (Paris et al., 2004; Zahir et al., 2015; Shadab et al., 2017). Cell cycle arrest is most likely the outcome of DNA fragmentation (Paris et al., 2004). Cell cycle analysis data suggested that sesamol treatment increased the population of promastigotes in G_0_/G_1_ phase. In untreated control, only 38.5% of the parasites were observed in G_0_/G_1_ phase of the cell cycle. It was further increased to 69.9 and 77% at IC_50_ and 2×IC_50_ concentrations, respectively. These results suggested that parasites were inhibited in G_0_/G_1_ phase in spite of entering into S-phase upon sesamol treatment (Zahir et al., 2015). Similar effects had been observed by other antileishmanial molecules such as berberine chloride, Spinigerin, and β-sitosterol, where significant populations of parasites had been arrested in G_0_/G_1_ phase of the cell cycle (Sardar et al., 2013; De Sarkar et al., 2019; Pramanik et al., 2020).

During apoptosis, several morphological changes were observed like cell shrinkage, PS externalization, cell cycle arrest, cell membrane blebbing, DNA fragmentation, and depolarization of mitochondrial membrane potential (Sardar et al., 2013; Hans et al., 2017). Translocation of PS in the outer leaflet of the membrane is the key feature of apoptosis-like cell death in the parasite. It had been reported that many secondary metabolites from plants like apigenin, ferulic acid, gallic acid, and flavonoids induced antileishmanial activity by inducing apoptosis in parasites (Antwi et al., 2019; Mustafa et al., 2021). We observed a similar mechanism of apoptosis in *L. donovani* promastigotes treated with sesamol. We observed profound induction of PS externalization at both IC_50_ and 2×IC_50_ doses. Our data showed that there is a substantial shifting of populations from early to late phase of apoptosis at increasing doses. Our findings strongly suggested that sesamol could be used as a potential drug candidate against VL.

## Conclusions

Sesamol restricted the growth and proliferation of *L. donovani* promastigotes in a dose-dependent manner. Sesamol potentially reduced intracellular parasite load in infected macrophages without showing toxicity towards host macrophages. The leishmanicidal effect of sesamol was mediated by apoptosis-like cell death mechanism as evidenced by cell cycle arrest, PS externalization, *in situ* DNA nicking, loss of mitochondrial membrane potential, and ROS generation. Hence, sesamol could be used as therapeutic herbal candidate for the treatment of VL after further validations.

## Data Availability Statement

The raw data supporting the conclusions of this article will be made available by the authors, without undue reservation.

## Author Contributions

Conceptualization, methodology, and supervision: RA, MA, AH, MH, and AR. Data curation: RA. Formal analysis: RA. Funding acquisition: AR, ASA, and MZA. Investigation: RA, SKA, ST, FR, and AR. Project administration: AR and MH. Writing original draft: RA and AR. Reviewing and editing: RA, ST, AH, MA, ASA, MZA, and MH. All authors contributed to the article and approved the submitted version.

## Funding

The authors are thankful to the Ministry of AYUSH for funding [Z.28015/252/2016-HPC (EMR)-AYUSH-C]. The authors are also thankful to the Researchers Supporting Project number RSP 2021/132, King Saud University, Riyadh, Saudi Arabia.

## Conflict of Interest

A patent application (Ref 2021110440550; Appl No. TEMP/E-1/50485/2021-DEL) was filed requesting a grant of Indian patent of the study.

## Publisher’s Note

All claims expressed in this article are solely those of the authors and do not necessarily represent those of their affiliated organizations, or those of the publisher, the editors and the reviewers. Any product that may be evaluated in this article, or claim that may be made by its manufacturer, is not guaranteed or endorsed by the publisher.
